# Fkh1 and Fkh2 Bind Multiple Chromosomal Elements in the *S. cerevisiae* Genome with Distinct Specificities and Cell Cycle Dynamics

**DOI:** 10.1371/journal.pone.0087647

**Published:** 2014-02-04

**Authors:** A. Zachary Ostrow, Tittu Nellimoottil, Simon R. V. Knott, Catherine A. Fox, Simon Tavaré, Oscar M. Aparicio

**Affiliations:** 1 Molecular and Computational Biology Program, University of Southern California, Los Angeles, California, United States of America; 2 Department of Biomolecular Chemistry, University of Wisconsin Medical School, Madison, Wisconsin, United States of America; Florida State University, United States of America

## Abstract

Forkhead box (FOX) transcription factors regulate a wide variety of cellular functions in higher eukaryotes, including cell cycle control and developmental regulation. In *Saccharomyces cerevisiae*, Forkhead proteins Fkh1 and Fkh2 perform analogous functions, regulating genes involved in cell cycle control, while also regulating mating-type silencing and switching involved in gamete development. Recently, we revealed a novel role for Fkh1 and Fkh2 in the regulation of replication origin initiation timing, which, like donor preference in mating-type switching, appears to involve long-range chromosomal interactions, suggesting roles for Fkh1 and Fkh2 in chromatin architecture and organization. To elucidate how Fkh1 and Fkh2 regulate their target DNA elements and potentially regulate the spatial organization of the genome, we undertook a genome-wide analysis of Fkh1 and Fkh2 chromatin binding by ChIP-chip using tiling DNA microarrays. Our results confirm and extend previous findings showing that Fkh1 and Fkh2 control the expression of cell cycle-regulated genes. In addition, the data reveal hundreds of novel loci that bind Fkh1 only and exhibit a distinct chromatin structure from loci that bind both Fkh1 and Fkh2. The findings also show that Fkh1 plays the predominant role in the regulation of a subset of replication origins that initiate replication early, and that Fkh1/2 binding to these loci is cell cycle-regulated. Finally, we demonstrate that Fkh1 and Fkh2 bind proximally to a variety of genetic elements, including centromeres and Pol III-transcribed snoRNAs and tRNAs, greatly expanding their potential repertoire of functional targets, consistent with their recently suggested role in mediating the spatial organization of the genome.

## Introduction

Forkhead Box (Fox) transcription factors comprise a large and diversified family of DNA binding proteins that function in a wide range of processes from yeast to humans, including cell cycle control, development, stress response, and apoptosis (reviewed in [1]). Common to these proteins is the Forkhead DNA-Binding Domain (Fkh-DBD) that binds DNA as a monomer through a conserved helix-turn-helix motif variant, known as a winged-helix. The Fkh-DBD typically recognizes a conserved core DNA sequence (RYMAAYA) with flanking nucleotides providing additional DNA sequence specificity for different Fkh-DBDs. In animals, Fox proteins have been characterized as pioneer transcription factors for their intrinsic ability to bind with sequence specificity to DNA within a compacted, nucleosomal context, and to remodel chromatin for transactivator accessibility and gene activation. Additionally, Fox proteins act in gene activation and repression through mechanisms involving recruitment of co-activators or co-repressors, including chromatin modifiers [1].

In *Saccharomyces cerevisiae*, four proteins contain a Fkh-DBD, including Fkh1, Fkh2, Hcm1, and Fhl1 (reviewed in [2]). Fhl1 has diverged substantially and binds unrelated DNA sequence(s). Hcm1 regulates the expression of a set of genes expressed during S-phase, including Fkh1 and Fkh2 [3]. Fkh1 and Fkh2 share the greatest sequence similarity with each other and recognize similar DNA sequences, which are largely distinct from those recognized by Hcm1 [3]–[13]. Fkh1 and Fkh2 also share a ForkHead-Associated (FHA) domain, a phosphothreonine-binding motif, while Fkh2 contains an additional C-terminal domain [6], [12]. Fkh1 and Fkh2 regulate a set of ∼33 genes, referred to as the *CLB2*-cluster, which are expressed during late S/G2-phase to regulate subsequent mitotic events [11].

Combined deletion of *FKH1* and *FKH2* severely diminishes expression of *CLB2*-cluster genes and induces pseudohyphal growth, normally a starvation response, whereas deletion of either gene alone has less severe effects on *CLB2*-cluster expression and does not cause pseudohyphal growth [6], [8], [11], [14], [15]. Thus, Fkh1 and Fkh2 can partially complement loss of each other’s function. However, the phenotypes of the single deletions are different on *CLB2*-cluster gene expression, with *FKH1* deletion being defective in transcriptional repression during G1-phase and *FKH2* deletion being defective in timing and peak transcriptional activation levels during late-S/G2 [6], [8], [11], [14]. Both proteins are thought to participate in *CLB2*-cluster gene repression, however, Fkh2, but not Fkh1, exhibits cooperative DNA-binding interaction with transcription factor Mcm1 that is key to transcriptional activation [5], [16]. In addition, Clb5-Cdk1-mediated phosphorylation of the unique C-terminus of Fkh2 promotes interaction with transcription factor Ndd1 that is reinforced by binding of Clb2-Cdk1-phosphorylated Ndd1 with the FHA domain of Fkh2, culminating in transcriptional activation [17]–[19]. *NDD1* is essential for *CLB2*-cluster gene activation and its deletion is lethal; however, this lethality is suppressed by deletion of *FKH2*, but not *FKH1*, consistent with the idea that Ndd1 overcomes repression by Fkh2 [15]. The interactions of Fkh2 with Mcm1 and Ndd1 have led to a greater focus in previous studies on Fkh2 rather than on Fkh1, and hence, how Fkh1 normally participates in *CLB2*-cluster regulation is less clear.

In contrast, *FKH1* has been uniquely implicated in regulation of mating-type switching (reviewed in [20]). Mating-type switching in budding yeast involves repair of a dsDNA break targeted to the *MAT* locus, resulting in a gene conversion event at *MAT*. The break is repaired by homologous recombination using one of two homologous donor mating-type alleles (***a*** or ***α***) on either distal arm of the chromosome. *Mat* ***a*** cells preferentially (∼95%) use *HML* ***α*** versus *HMR* ***a*** as the donor locus, resulting in a mating-type switch. This preference acts through a Recombination Enhancer (*RE*) element near *HML* ***α*** that binds Fkh1. Deletion of the *RE* or *FKH1*, but not *FKH2*, eliminates donor preference, and tethering of the Fkh1-FHA domain in place of the *RE* is sufficient to restore donor preference [21]–[23]. Thus, Fkh1 regulates the physical interaction between chromosomally distal DNA sequences.

More recently, *FKH1* and *FKH2* were reported to regulate replication origin timing through a mechanism also involving long-range chromosomal interactions resulting in clustering of early-firing origins [24]. Combined deletion of *FKH1* and *FKH2* alters the replication timing of most of the earliest- and latest-firing replication origins throughout the genome. Early origins that are delayed in *fkh1*Δ *fkh2*Δ cells (referred to as Fkh-activated origins) are locally enriched for Fkh1 and/or Fkh2 (Fkh1/2) consensus binding sequences, and deletion of these consensus binding sequences near an early origin deregulates its timing. Deletion of *FKH1* alone has a more modest effect, with ∼50 replication origins (early and late) detectably altered, while deletion of *FKH2* alone has no effect. Thus, *FKH1* appears to play the primary role in regulating replication origin timing while *FKH2* can partially substitute for *FKH1* in this function. The basis for this difference remains to be elucidated.

Previous studies of Fkh1 and Fkh2 chromatin binding using chromatin immunoprecipitation analyzed by DNA microarray (ChIP-chip) combined with analysis of consensus sequence conservation revealed a few hundred genomic binding loci for each protein [4], [7], [13]. However, these datasets did not report binding of Fkh1 or Fkh2 at many Fkh-activated origins, despite the recently reported enrichment of consensus binding sequences near these origins, suggesting that the existing data are incomplete. Indeed, the previous ChIP-chip study used early microarray technology with coverage of intergenic regions only, in most cases by a single cDNA probe per intergenic region. In addition, the previous study analyzed unsynchronized cell populations, which might miss cell cycle-regulated binding. We wished to generate more comprehensive and higher-resolution binding data for Fkh1 and Fkh2, and examine cell cycle regulation. Given the improvement in microarray platforms, instruments and reagents available for ChIP-chip studies, we undertook a new analysis of Fkh1 and Fkh2 binding. Our results indicate highly abundant binding of Fkh1 and Fkh2 throughout the genome with many shared and unique binding loci. Nucleosomal architecture differs at loci unique to Fkh1 versus loci that also bind Fkh2. We also observe cell cycle regulation of binding in the proximity of specific elements such as replication origins, and observe robust association with a variety of other genetic elements not previously reported, including RNA Pol III-transcribed genes. These findings provide an expanded map of Fkh1 and Fkh2 chromatin binding, provide novel insight into origin regulation, and suggest novel roles for Fkh1 and Fkh2 in genome regulation.

## Results

### An Expanded Map of Fkh1 and Fkh2 Binding to the *S. cerevisiae* Genome

**Figure 1:**
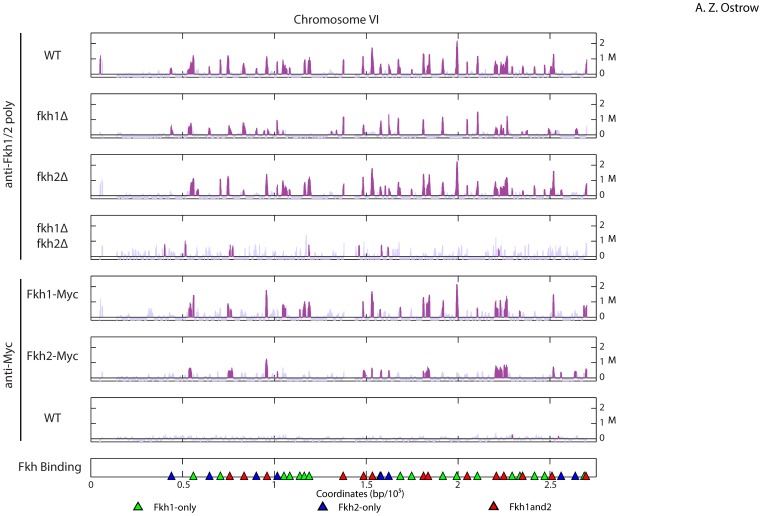
Genome-wide analysis of Fkh1 and Fkh2 chromatin binding. Plots show averaged ChIP-chip signal (M) from three experimental replicates along chromosome VI, with enriched regions plotted in purple. The antibody and strain genotype used for each experiment are indicated to the left of each panel; the corresponding strains from top to bottom are: CVy43, ZOy1, CVy138, CVy139, ZOy3, ZOy4, and CVy43. Triangles on the bottom panel indicate the position of determined binding sites as described in the text, color-coded by classification.

To assess the genome-wide distribution of Fkh1 and Fkh2, we performed ChIP-chip using several immunologic approaches. First, we used a polyclonal antibody that immunoprecipitates Fkh1 and Fkh2 (herein referred to as “anti-Fkh1/2 poly”) and carried out experiments in wild type (*WT*) and *fkh1*Δ *fkh2*Δ (control) strains. To validate and supplement these results, we also performed the analysis with anti-MYC monoclonal antibody in *WT* strains expressing C-terminally epitope-tagged Fkh1 (Fkh1-Myc9), Fkh2 (Fkh2-Myc13), and an untagged (control) strain. Experiments were performed in triplicate and analyzed with tiling microarrays covering unique sequences of the *S. cerevisiae* genome (one ∼60 bp oligonucleotide probe every ∼80 bp of unique sequence). Data from individual replicates were analyzed to identify significantly enriched regions (p≤0.05) having a minimum length of 500 bp (see **Methods**). Segments of these enriched regions that overlapped by at least 500 bp in at least two replicates were deemed “bound loci”, while any such regions overlapping substantially (≥ 50% of length) with regions deemed bound in the control strains (*fkh1*Δ *fkh2*Δ for anti-Fkh1/2 poly and untagged for anti-Myc) were excluded from the set. Plots of the data across chromosome VI show the average from the three replicates of each experiment with bound loci colored (Fig. 1; plots of all chromosomes are presented in Fig. S1).

**Figure 2:**
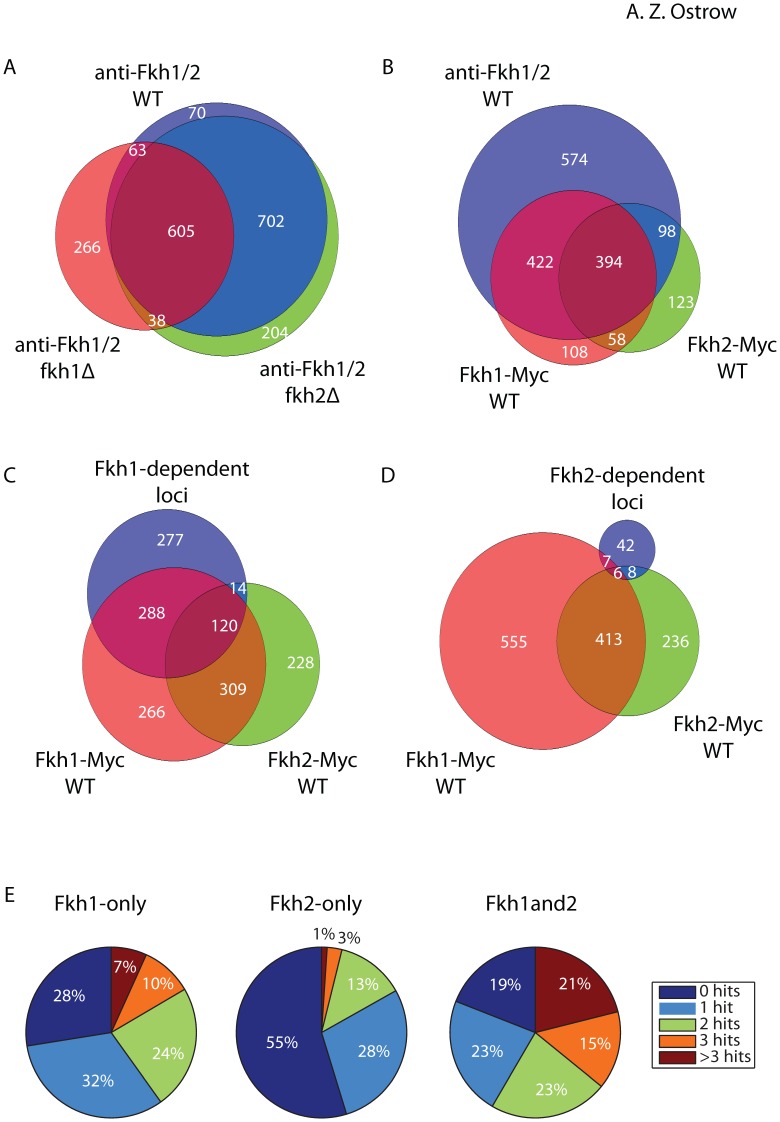
Correlation of Fkh1 and Fkh2 binding sites identified in different experiments. **A–D**) Venn diagrams show overlap between binding regions identified and/or categorized in different experiments. The area of the circle representing each group and the degree of intersection between groups are proportional to the number of binding loci in each group and degree of intersection, respectively. Discrepancies in number of total binding loci corresponding to datasets between the different Venn diagrams result from the method for calculating intersection between the sets (see **Methods S1**). **E**) Pie charts show the percentage of binding loci in each group for which the indicated number of matches to Fkh1 and/or Fkh2 consensus binding site(s) were identified. Because the values were rounded to the nearest whole number, the sum of percentages in two of the pie charts exceeds 100%.

Analysis with anti-Fkh1/2 poly identified 1503 Fkh1 and/or Fkh2 (Fkh1/2)-bound loci that were not detected in the control *fkh1*Δ *fkh2*Δ cells (Table S1). To investigate the dependence of these bound loci on Fkh1 and Fkh2, we performed ChIP-chip on *fkh1*Δ and *fkh2*Δ strains with anti-Fkh1/2 poly (Fig. 1, Table S1). We analyzed the resulting binding maps to identify overlapping regions (see **Methods**), which are indicated in the corresponding intersection of the Venn diagram (Fig. 2A). Focusing on the intersection of the *WT* with the *fkh1*Δ and *fkh2*Δ sets, 702 bound loci in *WT* and *fkh2*Δ cells were not bound in *fkh1*Δ cells, defining these as Fkh1-dependent loci and suggesting these loci specifically bind Fkh1 (Fig. 2A). 63 sites bound in *WT* and *fkh1*Δ cells were not bound in *fkh2*Δ cells, defining these as Fkh2-dependent loci and suggesting that these sites specifically bind Fkh2. The remaining 605 loci are defined as Fkh1/2-dependent loci, suggesting that these sites can bind both Fkh1 and Fkh2, either simultaneously or in the absence of the other.

Analysis with anti-Myc identified 1013 Fkh1-Myc- and 700 Fkh2-Myc-bound loci, which were not detected in the untagged strain (Table S1). These sets showed substantial overlap with the Fkh1/2-poly set, with 81% of the Fkh1-Myc and 70% of the Fkh2-Myc bound loci intersecting with the Fkh1/2 poly set, while the union of Fkh1-Myc and Fkh2-Myc sets intersected with 61% of the larger Fkh1/2 poly set (Fig. 2B). The Fkh1-Myc and Fkh2-Myc sets also showed substantial overlap with each other, with 452 loci exhibiting binding to both proteins. An additional 530 loci bound Fkh1-Myc specifically, and 221 loci bound Fkh2-Myc specifically.

To test these inferred specificities, we examined Fkh1-Myc and Fkh2-Myc binding at Fkh1- and Fkh2-dependent loci determined in the experiments with anti-Fkh1/2 poly. Fkh1-dependent loci showed greater overlap with Fkh1-Myc (58%) than Fkh2-Myc (19%) loci (Fig. 2C), whereas a more balanced proportion of all Fkh1/2 poly loci overlapped with Fkh1-Myc (55%) and Fkh2-Myc (34%) loci (Fig. 2B), consistent with specific or preferential binding of Fkh1 to the set of Fkh1-dependent loci. In contrast, the comparatively small number of Fkh2-dependent loci showed similar overlap with Fkh2-Myc (22%) and Fkh1-Myc (21%) loci (Fig. 2D). Overall, the multiple approaches, use of controls, and good overlap between datasets suggests we have generated robust Fkh1 and Fkh2 binding data. We consolidated the data into three, non-overlapping sets for further analysis, yielding: 828 Fkh1-only loci, which were only detected to bind Fkh1, 285 Fkh2-only loci, which were only detected to bind Fkh2, and 541 Fkh1and2 loci, which were detected to bind Fkh1 and Fkh2 (see **Methods, Table S2**).

To examine these sets of Fkh1 and Fkh2 binding loci further, we searched for Fkh1 and Fkh2 consensus binding sequences within the called regions. Using previously reported position-weight matrices of Fkh1 and Fkh2 consensus sequences [9], we determined coordinates for Fkh1 and Fkh2 consensus sequences in the yeast genome (Table S3). The Fkh1 and Fkh2 consensus sequences are very similar to each other, so we searched for the presence of either one, within each set of bound loci. 72%, 45%, and 81% of the Fkh1-only, Fkh2-only, and Fkh1and2 bound loci, respectively, contained at least one Fkh1/2 consensus sequence match (Fig. 2E).

### Fkh1 and Fkh2 are Associated with Distinct Chromatin Architectures

**Figure 3:**
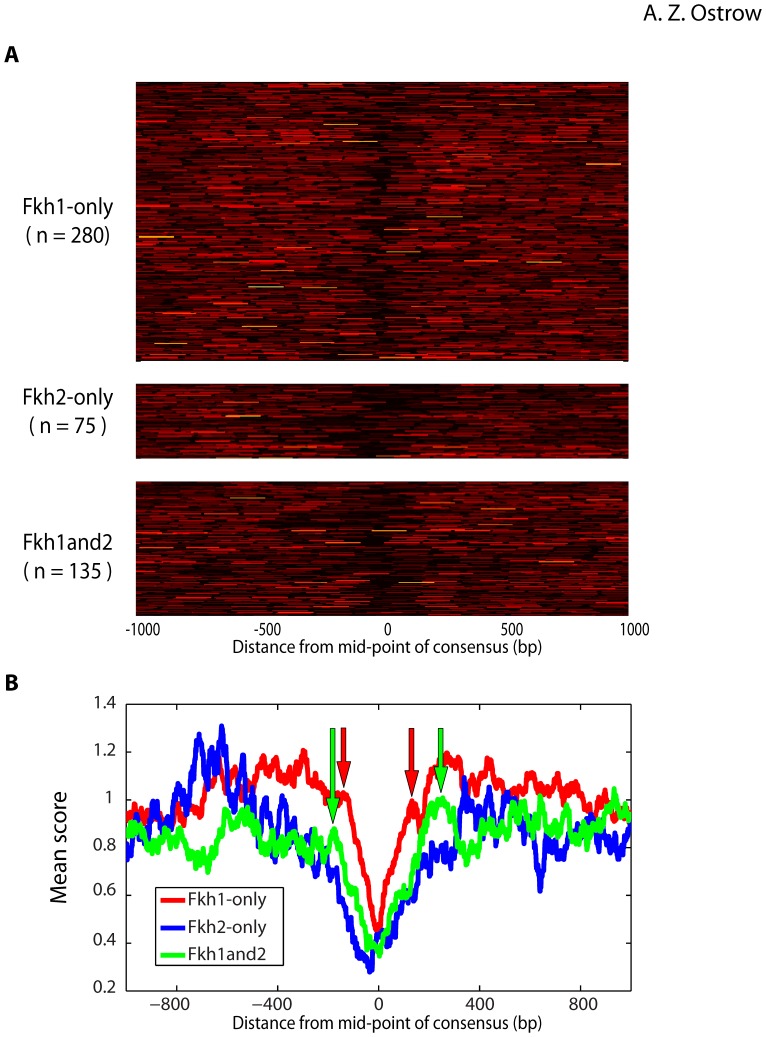
Distinct nucleosome positioning at Fkh1-only loci versus loci that bind Fkh2. **A)** The heat maps show density of MNase-protected sequences (Eaton et al 2010) for 2 kb regions centered on Fkh1/2 consensus sequences within enriched regions that have only a single Fkh1/2 consensus sequence. **B)** Averaged signal intensities from **(A)** are plotted. Arrows indicate the positions used to estimate length of nucleosome-depleted regions reported in the **Results**.

Fkh1 and Fkh2 have been implicated in the regulation of chromatin structure through the recruitment of chromatin modifiers and remodelers [25]–[27], so we examined the chromatin structure associated with Fkh1 and Fkh2 binding. To achieve base-pair resolution necessary to compare binding with nucleosome positioning, we examined Fkh1- and Fkh2-bound loci containing a single Fkh1/2 consensus sequence(s) and aligned these sequences with a published map of nucleosome positions [28]. We plotted the nucleosome density in a 2 kb region surrounding each consensus sequence bound by Fkh1-only, Fkh2-only, and Fkh1and2 loci, as separate sets (Fig. 3A). The data show differences in the nucleosome densities associated with these bound loci, with Fkh1-only loci localizing to narrower nucleosome-depleted regions than Fkh2-only and Fkh1and2 loci. We consolidated the data into an average nucleosome density profile for each set and plotted the profiles together for comparison (Fig. 3B). Estimation of the size of the nucleosome-depleted regions indicates a length of ∼400 bp at Fkh1and2 loci versus ∼275 bp at Fkh1-only loci, a difference of approximately one nucleosome.

### Fkh1 and Fkh2 Binding at Regulated Genes

**Figure 4:**
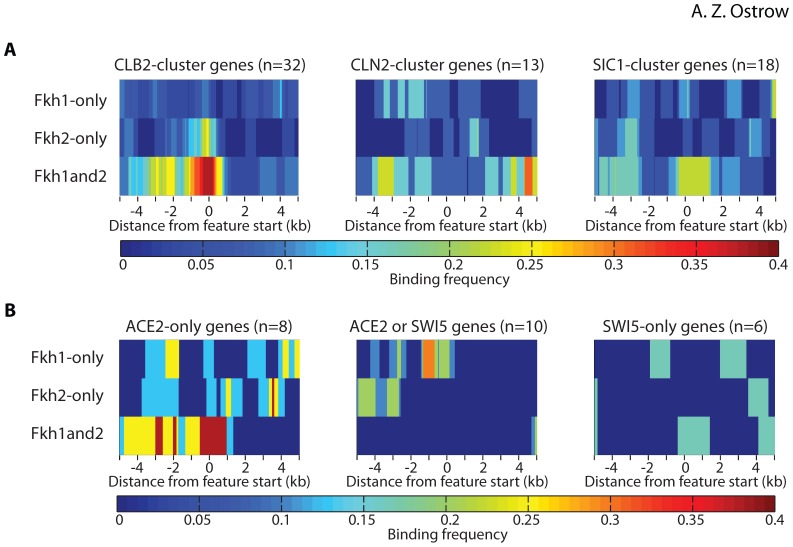
Fkh1 and Fkh2 binding with target genes. Heat maps show 10 kb regions of summed binding data for the indicated types of binding loci (Fkh1-only, Fkh2-only, Fkh1and2) surrounding the groups of features indicated above the heat map. The color represents the frequency of enriched binding sequences called for each group of features, amongst total number of features (n) included in each group. **A**) Fkh1 and Fkh2 enrichment frequencies surrounding *CLB2-*, *CLN2-*, and *SIC1*-cluster genes are plotted as separate groups, with the respective ORFs aligned by their start codons at coordinate 0, with transcription toward positive coordinates to the right. **B**) Fkh1 and Fkh2 enrichment frequencies surrounding Ace2-only-regulated genes, Ace2- or Swi5-regulated genes, and Swi5-only-regulated genes are plotted with the ORFs aligned and oriented as in (**A**).

Next, we examined the Fkh1 and Fkh2 binding data at genes previously reported to be under Fkh1/2 regulation. We generated heat maps of Fkh1-only, Fkh2-only, and Fkh1and2 binding frequency for 10 kb regions centered and oriented on the start codons of 32 *CLB2*-cluster genes and, for comparison, two additional groups of co-regulated genes: 13 “*CLN2*-cluster” genes expressed in late G1-phase and 18 “*SIC1*-cluster” genes expressed in late M-early G1-phase (Fig. 4A) [29]. The heat maps show enrichment of Fkh1 and Fkh2 over the promoter regions of *CLB2*-cluster genes, with 38% of these regions binding both proteins, an additional 21% binding only Fkh2, and an additional 8% binding only Fkh1. In comparison, Fkh1 and Fkh2 were not enriched over the promoters of the *CLN2*-cluster genes, as expected. Interestingly, some enrichment of Fkh1 and Fkh2 was apparent over *SIC1*-cluster genes, which is consistent with Fkh1 and Fkh2 acting as anti-activators of a subset of *SIC1*-cluster genes resulting in their activation by Ace2 but not by Swi5 [27]. To examine this more closely, we divided the *SIC1* gene cluster into subsets activated by transcription factor Ace2 only, Swi5 only, or either factor, and generated heat maps of Fkh1 and Fkh2 binding frequencies (Fig. 4B). The results show occupancy of Fkh1 and Fkh2 at 38% of Ace2-only genes, but little to no occupancy at other *SIC1*-cluster genes, confirming that Fkh1 and Fkh2 specifically bind Ace2-only genes [27]. These findings demonstrate that our data recapitulate known features of Fkh1 and Fkh2 binding.

### Fkh1 and Fkh2 Binding at Replication Origins

**Figure 5:**
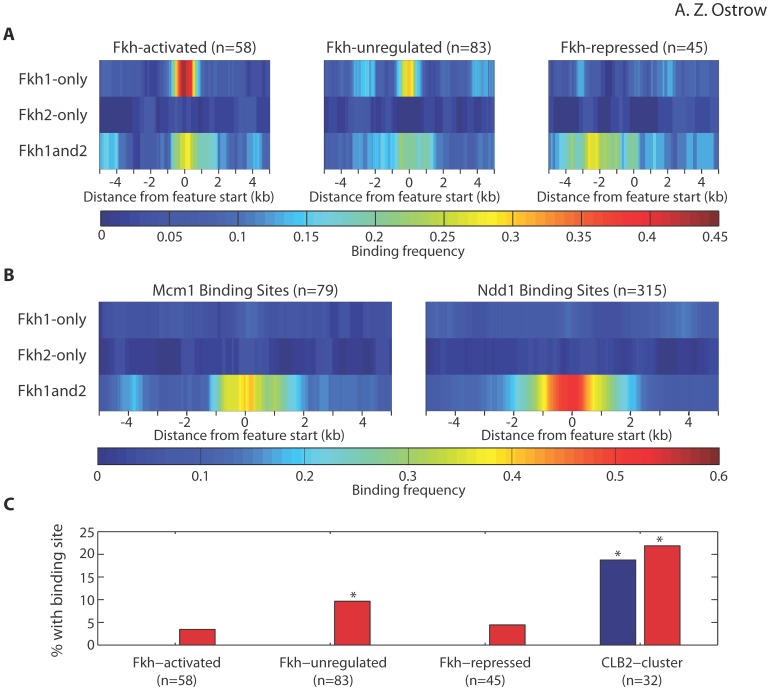
Fkh1 and Fkh2 binding with replication origins. (**A–B**) Heat maps show 10 kb regions of summed binding data for the indicated types of binding loci (e.g.: Fkh1-only, Fkh2-only, Fkh1and2) surrounding the groups of features indicated above the heat map. The color represents the frequency of enriched binding sequences called for each group of features, amongst total number of features (n) included in each group. **A**) Fkh1 and Fkh2 enrichment frequencies surrounding Fkh-activated, Fkh-unregulated, and Fkh-repressed origins are plotted with each group aligned and oriented at coordinate 0 by each origin’s ARS consensus sequence (ACS). **B**) Fkh1 and Fkh2 enrichment frequencies are plotted around Mcm1 and Ndd1 binding sites, which are aligned and oriented by Mcm1 and Ndd1 consensus sequences, respectively. **C**) The graph shows the percentage of each element class having an Mcm1 or Ndd1 binding site within 500 bp. Asterisks indicate values significantly greater than expected on a random basis at p<0.01 (see Methods S1).

Fkh1/2 were recently identified as regulators of the initiation timing of replication origins throughout the budding yeast genome [24], [30]. In *fkh1*Δ *fkh2*Δ cells, the initiation of many early origins is delayed, and these origins are locally enriched for Fkh1/2 consensus binding sequences. For a few tested origins, Fkh1/2 binding sequences in *cis* were shown to be essential for regulation of the proximal origin. However, previous ChIP-chip analysis did not report Fkh1/2 binding at many Fkh-regulated origins [4], [7], [13], suggesting that Fkh1/2 might act over longer distances to regulate some origins. To examine the Fkh1- and Fkh2-bound loci we have identified in relation to replication origins, we divided origins (termed ARS in yeast) into three groups defined by their change in origin activity in *fkh1*Δ *fkh2*Δ cells in our previous study: Fkh-activated origins, which showed reduced early firing, Fkh-repressed origins, which showed increased early firing, and Fkh-unregulated origins, which showed no significant change in early firing [24]. For each set of origins, we generated heat maps representing the frequency of Fkh1-only, Fkh2-only and Fkh1and2 bound loci for a 10 kb region centered and oriented on the ARS Consensus Sequence (ACS), which is the essential origin-defining sequence (Fig. 5A). Fkh-activated origins are enriched for proximal Fkh1 binding, with 42% of these origins associated with Fkh1-only loci and an additional 27% associated with Fkh1and2 loci, while only 2% are associated with Fkh2-only loci. Fkh-unregulated origins are also enriched for Fkh1, with 31% of these origins associated with Fkh1-only loci and 21% associated with Fkh1and2 loci. Only 11% of Fkh1-only and no Fkh2-only loci are associated with Fkh-repressed origins, however, 20% of Fkh-repressed origins are associated with Fkh1and2 binding loci. These results are consistent with our previous demonstration that Fkh1/2 consensus binding sequences are enriched near Fkh-activated origins and required for their regulation, whereas Fkh1/2 consensus sequences are depleted near Fkh-repressed origins [24]. However, these results also suggest that Fkh1/2 binding is not sufficient to establish Fkh-activation or that Fkh-unregulated origins are associated with factors that oppose Fkh-origin regulation (see **Discussion**). The results further suggest that Fkh-repression of origins may in some cases derive from direct binding by Fkh1/2.

The predominance of Fkh1 over Fkh2 binding near origins was consistent with our previous finding that *fkh1*Δ cells deregulate origin timing whereas *fkh2*Δ cells do not (see **Introduction**). However, our previous study also showed that *fkh1*Δ *fkh2*Δ cells deregulate many additional origins than *fkh1*Δ cells, suggesting a primary role for Fkh1 in origin timing regulation and a secondary role for Fkh2 [24]. Given our previous findings that both Fkh1 and Fkh2 consensus binding sequences are enriched near Fkh-activated origins, the preference for Fkh1 binding indicates the existence of additional determinants of Fkh1 versus Fkh2 binding specificity. Possible candidates for determining Fkh1 versus Fkh2 binding specificity are Mcm1 and Ndd1. *In vitro,* Mcm1 binds cooperatively with Fkh2, but not Fkh1, to DNA sequences containing closely juxtaposed Fkh1/2 and Mcm1 consensus binding sequences [5], [16]. *In vivo*, Fkh2 recruits Ndd1 to *CLB2*-cluster gene promoters through interactions involving the unique C-terminus of Fkh2 [18], [19].

To examine the relationship of Mcm1 and Ndd1 with Fkh1 and Fkh2 binding, we plotted Fkh1-only, Fkh2-only, and Fkh1and2 binding loci for 10 kb regions centered on 79 Mcm1 and 315 Ndd1 binding sites, which were previously reported to bind the respective protein in ChIP experiments and contain a recognizable consensus sequence for the respective protein [4], [7]. The heat maps show strong enrichment of Fkh1and2-bound loci proximal to Mcm1 binding sites, with 41% of Mcm1 binding sites overlapping with a Fkh1and2 locus. A few Fkh1-only and almost no Fkh2-only loci were associated with Mcm1 binding sites (Fig. 5B). Ndd1 exhibited a similar pattern of association, with 52% of Ndd1 binding sites proximal to Fkh1and2 loci, 13% of Fkh1-only loci and almost no Fkh2-only loci are proximal to Ndd1 binding (Fig. 5B). Because Fkh-activated replication origins are associated predominantly with Fkh1-only binding loci, this result implies that neither Mcm1 nor Ndd1 associates with most Fkh-activated origins. We tested this directly by searching for Mcm1 and Ndd1 binding sites proximal to replication origins, and for comparison, to *CLB2*-cluster genes. The results show no instances of Mcm1 binding sites within 500 bp of any of the replication origin classes, whereas 19% of *CLB2*-cluster genes are within 500 bp of an Mcm1 binding site (Fig. 5C). Like Mcm1, Ndd1 binding sites are also enriched at *CLB2*-cluster genes, with 22% of *CLB2*-cluster genes proximal to an Ndd1 site. In contrast to Mcm1, however, Ndd1 binding sites are associated with 10% of Fkh-unregulated origins, representing significant enrichment with this origin class, and with 3% and 4% of Fkh-activated and Fkh-repressed origins, respectively (Fig. 5C). These results suggest that recruitment of Ndd1 to replication origins might counteract Fkh1/2-regulation of origin function (see **Discussion**).

### Other Genetic Elements Associated with Fkh1 and Fkh2 Binding

**Figure 6:**
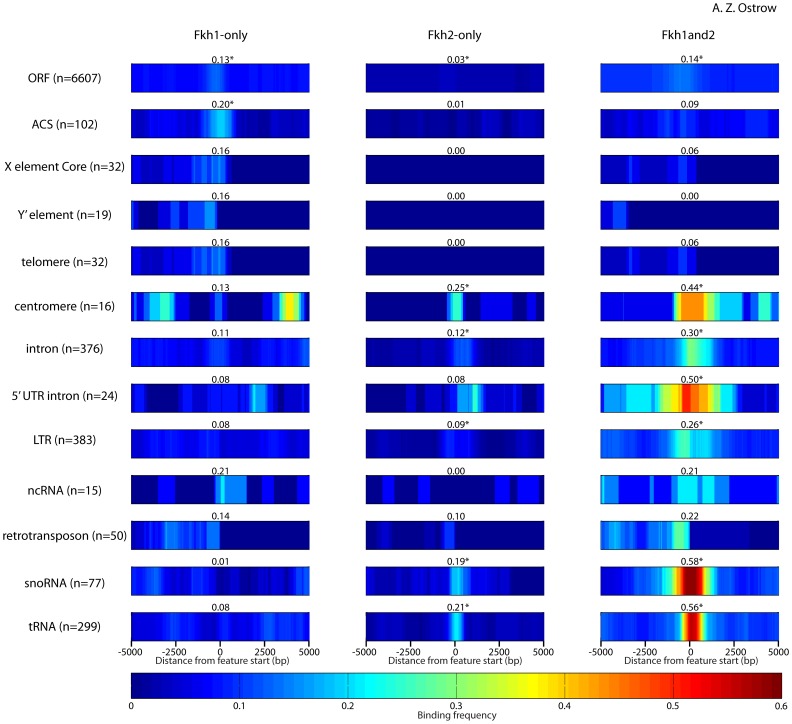
Analysis of Fkh1 and Fkh2 binding proximal to various genetic elements. Fkh1 and Fkh2 enrichment frequencies surrounding different classes of genetic elements are oriented and aligned at coordinate 0 according to the first base position of each element. The maximum frequency reached within 100(500 bp for Y′) of coordinate 0 is indicated above each heat map. The asterisk indicates significant enrichment (p<0.001) near coordinate 0 (see **Methods**).

To determine whether Fkh1 and Fkh2 bind and potentially regulate other genomic elements, we plotted Fkh1 and Fkh2 binding loci near different sets of genomic elements (as defined in Saccharomyces Genome Database) (Fig. 6). Fkh1 and Fkh2 showed remarkable occupancy near several of these elements, with occupancy rates comparable to those at *CLB2*-cluster genes and Fkh-activated origins. As a group, ORFs show minor enrichment of Fkh1 or Fkh2 relative to flanking sequences. ARSs, telomeres, and subtelomeric X and Ý elements, are associated predominantly with Fkh1-only, with 15–20% of these elements proximal to a Fkh1-only locus. In contrast, centromeres, 5′ UTR introns, snoRNAs, and tRNAs are more frequently associated with Fkh1and2 binding loci, which are proximal to 40–60% of these elements; these elements show more modest levels of enrichment for Fkh2-only loci (see Table S4 for list of genes with Fkh1/2 enrichment upstream). Fkh1and2 binding loci are also proximal to 20–30% of introns, ncRNAs, retrotransposons, and dispersed long terminal repeats (LTRs). Interestingly, ncRNAs were associated with Fkh1-only binding loci at a similar frequency as with Fkh1and2 loci. These findings suggest that Fkh1 and Fkh2 have unrecognized roles in the regulation of Pol III-transcribed genes, intron processing, and centromere function.

### Cell Cycle Dynamics of Fkh1 and Fkh2 Binding

**Figure 7:**
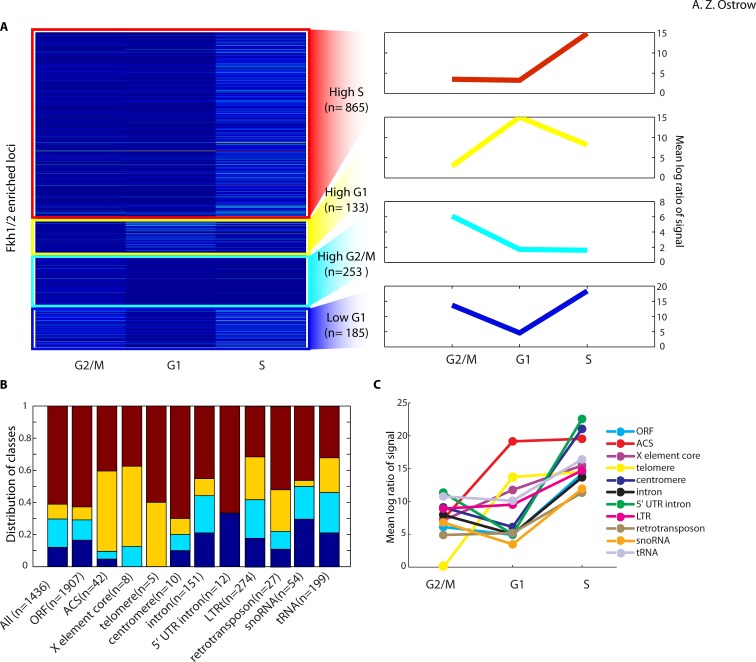
Cell cycle analysis of Fkh1 and Fkh2 binding. **A**) The k-means cluster-gram on the left shows the average signal intensity of individual binding loci across the three experiments, divided into four groups, which was found to account well for the data. The graphs to the right of each cluster show the averaged signal of all sites in the cluster. **B**) For each class of genetic element indicated, the number of proximal (+/−500 bp) Fkh1 and Fkh2 binding loci in each of the four clusters in (**A**) was counted to determine the distribution of these binding sites amongst the four clusters. The colors in the graph correspond to the colors of the four clusters in (**A**). **C**) The average signals of Fkh1 and Fkh2 binding loci proximal (+/−500 bp) to the indicated genetic element class was determined and plotted for the three cell cycle points.

To gain further insight into the mechanisms that Fkh1/2 use to regulate genes and origins in the cell cycle context, we performed ChIP-chip of Fkh1 and Fkh2 with anti-Fkh1/2 poly in cells synchronized in G2/M with nocodazole, in late G1 with α-factor, and in early S with hydroxyurea. Data from these experiments corresponding to the Fkh1/2 binding loci identified above were subjected to k-means clustering analysis according to the binding patterns of individual loci across the three cell cycle stages (Fig. 7A, Table S2, see **Methods**). This analysis revealed four distinct clusters that can account for most of the data, with each cluster representing a distinct binding pattern across the cell cycle (Fig. 7A). The largest cluster of ∼865 binding loci, named “High-S”, shows higher binding in early S-phase and lower binding in G2/M and late G1. The High-G1 cluster shows higher binding in late G1 and lower binding in G2/M and early S. The High-G2/M cluster shows higher binding in G2/M and lower binding in late G1 and early S, while the Low G1 cluster shows lower binding in late G1 and higher binding in early S and G2/M.

To ascertain whether these cell cycle binding patterns are associated with specific functional classes of Fkh1/2 binding loci such as those associated with *CLB2*-cluster genes or replication origins, we determined the binding patterns of Fkh1/2 binding loci within 500 bp of specific classes of genomic features analyzed above (Fig. 7B). This analysis indicates that Fkh1/2 binding loci proximal to distinct genomic elements exhibit significantly distinct cell cycle patterns of Fkh1/2 binding (see **Methods**). For example, the High G1 binding pattern, which is the least frequent overall when all binding loci are considered, is the most frequent pattern associated with ARS and X elements, and is also significantly enriched at LTRs, ncRNAs, retrotransposons, tRNAs, and telomeres. The High G1 pattern is also depleted at snoRNAs. The Low G1 pattern, which is infrequent in the overall distribution, is significantly enriched at Introns, 5′ UTR Introns, snoRNAs, and tRNAs; this pattern is also depleted at ARSs, X elements and telomeres. The High G2/M pattern is modestly enriched at Introns, LTRs, and tRNAs, and is most notably depleted near ARSs. The High-S pattern, which is most frequent overall, is correspondingly depleted at most of the aforementioned elements that are enriched for another pattern. However, the High-S pattern is not depleted at binding loci proximal to ORFs, telomeres, centromeres, and 5′ UTR Introns.

To scrutinize the binding dynamics more directly at these genomic elements, we plotted Fkh1/2 binding profiles at loci specifically proximal to each set of elements (Fig. 7C). The plots show distinct binding patterns associated with different element types. For example, ARSs and telomeres show lower signals in G2/M and sharply higher signals in late G1 and in early S. In contrast, centromeres and 5′ UTR Introns showed intermediate signals in G2/M decreasing in early G1 followed by strikingly higher signals in early S. The remaining elements also generally showed higher signals in early S compared with G2/M and early G1, however, the overall degree of fluctuation was somewhat lower. With the exception of the very low binding at telomeres in G2/M, binding levels show the greatest differences amongst elements in late G1.

**Figure 8:**
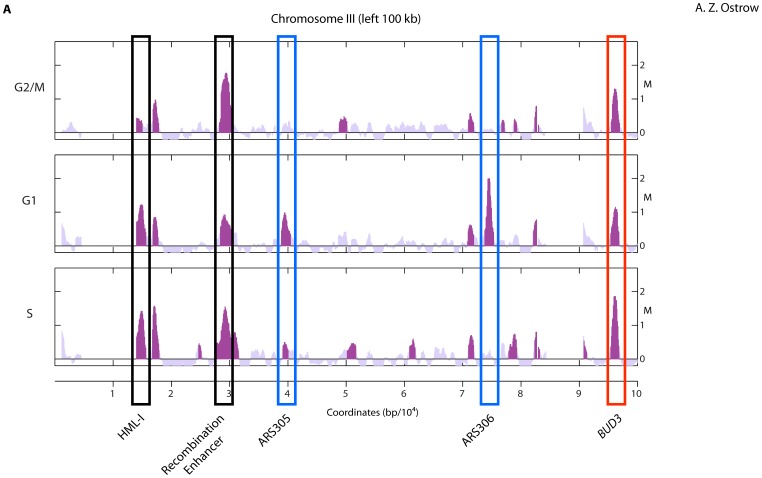
G1-specific binding of Fkh1/2 at Fkh-activated origins. Plots show averaged ChIP-chip signal from three experimental replicates along a 100 kb region of the left arm of chromosome III, with enriched regions plotted in purple. The cell cycle arrest for each experiment is indicated to the left of each panel. Boxed loci are discussed in the **Results**.

To examine Fkh1/2 binding at specific loci, particularly Fkh-activated origins, we plotted the cell cycle ChIP data for a 100 kb region of chromosome III (Fig. 8, see Fig. S2 for plots of all chromosomes). This region includes early-efficient origins *ARS305* and *ARS306*, the silent mating-type locus *HML*, the Recombination Enhancer (*RE*) for mating-type donor preference, and *BUD3*, a Fkh1/2-regulated *CLB2*-cluster gene, all of which are associated with Fkh1/2 binding. A previous study reported binding of Fkh1 and Fkh2 to *CLB2*-cluster target genes in late G1- and G2/M-synchronized cells, suggesting that Fkh1/2 bind constitutively to *CLB2*-cluster target genes [15]. In agreement with these previous reports, Fkh1/2 binding was strongly enriched at *BUD3* at all cell cycle times tested. In contrast, previous analysis of Fkh1 binding at the *RE* showed binding in G2/M but not in late G1 [31]. However, our data show binding of Fkh1/2 at all three cell cycle times, though we note a decreased signal in late G1. At *HML-I*, Fkh1/2 binding was detected at all cell cycle times, though the signal was decreased in G2/M. Unlike Fkh1/2 binding at all of these loci, however, Fkh1/2 binding at Fkh-activated origins *ARS305* and *ARS306* showed strong enrichment in G1-phase, but little or no enrichment in S- or G2/M-phases. These findings reveal a new dimension of Fkh1/2 regulation and support the notion that Fkh1/2 function through distinct mechanisms to regulate distinct classes of genetic elements.

## Discussion

### An Expanded Map of Fkh1 and Fkh2 Binding to the *S. cerevisiae* Genome

The recent discovery that Fkh1 and Fkh2 regulate replication initiation timing [24], [30], along with exciting new mechanistic insight into how Fkh1 regulates donor preference in mating-type switching [21], in addition to their well-established roles as transcription factors, have stoked new interest into these versatile regulators of the genome. A primary goal of this study was to gain a greater understanding of the relationship between Fkh1/2 binding and regulation of replication origins. Elucidating a more complete and dynamic map of Fkh1 and Fkh2 binding loci throughout the genome enabled robust, genome-scale analyses of these binding loci in relation to replication origins, as well as other functional genetic elements. We identified hundreds of novel binding loci for both proteins, including shared and specific loci. Analyses of these data showed binding to known binding loci and targets of regulation such as *CLB2*-cluster genes, serving to validate these results. These new genomic maps of Fkh1 and Fkh2 binding also provide a valuable resource for future genome-wide and locus-specific studies.

Our analysis of Fkh1/2 binding throughout the genome paints a somewhat different picture than previous studies [4], [13], with several-fold more binding loci, especially loci binding only Fkh1, identified here. To provide further confidence for our sets of identified binding loci, we searched for matches to Fkh1/2 consensus binding sequences. We found that a large majority of Fkh1-only and Fkh1and2 loci contained at least one consensus match within the enriched region, however, only slightly fewer than half of the Fkh2-only loci contained a match. We chose not to use the presence of a consensus sequence as a filter to reduce the number of called loci to avoid imposing this possible bias, as it remains possible that close matches to the consensus sequence were missed, or that Fkh1/2 binds some sequences independently of a consensus sequence. A related possibility is that binding loci lacking a consensus sequence represent sites of “indirect” binding (as coined by Bulyk and colleagues in [32]) where Fkh1/2 do not bind DNA directly but bind chromatin through interaction with other DNA-binding proteins.

The much larger number of Fkh1-only versus Fkh2-only loci suggests that Fkh2 binding is more specific or otherwise restricted. This might be explained by additional specificity provided by its interacting partners Mcm1 and/or Ndd1. Hence, it is surprising that Mcm1 and Ndd1 binding sites are located proximal to Fkh1and2 loci but not to Fkh2-only loci. This finding suggests that a different factor is responsible for the exclusive binding of Fkh2 at Fkh2-only loci. Whereas the more extensive nucleosome-depleted regions associated with Fkh1and2 binding loci may be related to Mcm1 and/or Ndd1 binding, this does not explain the similar nucleosomal structure observed at Fkh2-only loci, which are not associated with Mcm1 or Ndd1. Instead, the narrower nucleosome-depleted regions associated with Fkh1-only loci and the larger numbers of loci that bind Fkh1 (i.e., Fkh1-only and Fkh1and2 loci) suggest that Fkh1 is better able to access potential binding sequences in chromatin than Fkh2. A related possibility is that greater abundance of Fkh1 (1720 molecules/cell) versus Fkh2 (656 molecules/cell) results in a more restricted set of binding loci for Fkh2 [33]. Alternatively, Fkh1 and Fkh2 binding may regulate the remodeling of chromatin in distinct ways resulting in the observed differences. This is currently under investigation.

### Fkh1 and Fkh2 Binding at Replication Origins

In contrast to the high occupancy of both Fkh1 and Fkh2 at *CLB2*-cluster genes, Fkh-activated replication origins are most frequently bound by Fkh1 only, and whereas a minority of origins is also bound by Fkh2, almost none binds only Fkh2. These findings are consistent with the differential effects on individual origin function when either *FKH1* or *FKH2* is deleted [24]. These results also reinforce previous findings that Fkh1/2 act directly in *cis* to regulate origin function [24], [30]. Nevertheless, we did not detect Fkh1/2 binding near one-third of Fkh-activated origins, leaving open the possibility that the regulation of some origins occurs over a longer distance or indirectly. We also detected Fkh1, and to a lesser degree Fkh1 and Fkh2, binding at a fraction of origins in the Fkh-unregulated group. Some of these may represent *bona-fide* Fkh-activated origins within this set that did not reach the significance threshold to be classified as Fkh-activated in the previous study. However, another possibility is that additional chromatin regulators binding in the vicinity of these origins oppose Fkh1/2 function, resulting in their Fkh-unregulated phenotype. Indeed, the presence of Ndd1 binding sites near Fkh-unregulated origins may explain why some of these origins are Fkh-unregulated despite many of these origins being bound by Fkh1 and Fkh2.

The cell cycle-regulated association of Fkh1/2 with replication origins reported here is an important advance toward a complete understanding of the mechanism of Fkh1/2-regulation of origin timing. Previous studies have indicated that the establishment of the replication-timing program occurs in the M to early G1 period [34], [35]. More recent studies indicate that the selective recruitment of replication initiation factors to licensed origins during G1-phase determines early origin firing, and Fkh1/2 are required for this recruitment (reviewed in [36]). This strongly suggests that the G1-phase recruitment of Fkh1/2 is essential for initiation factor recruitment and is linked to the origin licensing cycle. This might involve interactions with protein(s) that license origins in early G1-phase such as Mini-Chromosome Maintenance proteins, and/or might involve regulation by CDK or DDK activities. Experiments are in progress to determine the mechanism of cell cycle-regulated binding of Fkh1/2 to replication origins.

### Novel Genetic Elements Associated with Fkh1 and Fkh2 Binding

A novel finding of this study is the association of Fkh1/2 with a large number of functional genetic elements, including centromeres, telomeres, transposable elements, introns and RNA Pol III-transcribed genes, suggesting a possible role for Fkh1/2 in regulating the function of these elements. Enrichment of Fkh1 upstream of tRNA genes has been previously reported [37]. The high Fkh1/2 occupancy at tRNAs and snoRNAs, which are transcribed by RNA Pol III is particularly intriguing given the known role of Fkh1/2 as a regulator of some Pol II-transcribed genes. Furthermore, Fkh1/2 are thought to regulate origin timing and mating-type donor preference by mediating long-range intra- and/or inter-chromosomal interactions (reviewed in [20], [36]), while highly expressed tRNAs aggregate into clusters surrounding the nucleolus (reviewed in [38]). It will be interesting to determine whether Fkh1/2 regulate tRNA clustering or expression. Similarly, the association of Fkh1/2 with transposable elements, centromeres and telomeres, all suggest a function in chromosomal organization.

The Fkh1/2 association with one or more of these element classes may reflect co-localization of two or more element classes where a single class is the functional target of Fkh1/2. A possible case is the enrichment of Fkh1 with telomeres and subtelomeric X and Y′ elements, which are associated with a high density of ARS elements [39], [40]. Thus, the binding of Fkh1 near subtelomeric origins likely explains their observed proximity to subtelomeric elements and telomeres. Although telomeres and subtelomeres are late-replicating, many of these regions become even later replicating in *fkh1*Δ *fkh2*Δ cells, consistent with Fkh1/2 regulating subtelomeric origins [24]. tRNAs and retrotransposons also co-localize with replication origins more frequently than expected at random [40]; however, this relationship probably does not explain the Fkh1/2 association with these elements because tRNAs and retrotransposons are primarily associated with Fkh1and2 binding loci whereas origins are primarily associated with Fkh1-only loci. Nevertheless, yeast transposable elements frequently co-localize with tRNAs and Pol III-transcribed genes so the association seen with these various elements may result from this co-localization. Given the much higher occupancy of Fkh1/2 at tRNAs and snoRNAs and the larger number of these elements compared with retrotransposons, we think it is more likely that the association with retrotransposons reflects functional Fkh1/2 binding near tRNAs and snoRNAs, rather than the converse. Whereas further studies will be required to elucidate fully the role(s) of Fkh1 and Fkh2 at these various elements, these remarkably robust associations strongly suggest that Fkh1 and Fkh2 have more global functions than previously appreciated. It remains to be seen whether the association of Fkh1 and Fkh2 with a broad array of genetic elements can be explained by a common mechanism involving higher-order chromatin organization.

## Methods

### Yeast Strains and Methods

All strains (see Table S5) are congenic with the W303 background, including *FKH1* and *FKH2 MYC*-tagged strains, Z1448 and Z1370 respectively, from the Young lab [4]. ZOy3 and ZOy4 were constructed by deletion of *BAR1* in strains Z1448 and Z1370, respectively, using *BamHI*-*BglII*-digested plasmid pΔbar1::URA3 with lithium acetate transformation [41]. Cells were grown at 23°C for all experiments and synchronized in late G1, early S, and G2/M by incubation for 3 h in 7.5 nM α-factor (Sigma, T6901), 200 mM hydroxyurea (Sigma, H8627), or 10 µg/mL nocodazole (Sigma, M1404), respectively. ChIP-chip experiments were performed as described previously [42], with the following modifications and reagents: chromatin was sheared to an average size of 300 bp using a Covaris S2 instrument; immunoprecipitations were performed with 9E10 (Covance, MMS150) at 1∶100 followed by pull-down with Protein G Dynabeads (Invitrogen, 10004D), or with anti-Fkh1/2 polyclonal antibody [43], which was pre-crosslinked to protein A-Sepharose 4B beads (Invitrogen, 10–1041), at 1∶40 (packed bed volume). Up to 10 ng immunoprecipitated (IP) and total DNA samples were subjected to whole genome amplification (Sigma, WGA2), followed by primer extension labeling with Cy5 and Cy3 end-labeled random nonamers, as described previously [42]. Cy5-labeled IP and Cy3-labeled total DNA samples were combined and hybridized to custom oligonucleotide tiling microarrays (Roche-Nimblegen, 124 k HX12) that tile one ∼60 bp oligonucleotide probe per ∼80 bp of unique genomic sequence; the Maui hybridization system and reagents (Roche) were used according to the manufacturer’s instructions, and image capture was performed using an Axon 4100A scanner.

### Microarray Data Analysis and Peak Calling

We used RINGO package (http://www.biomedcentral.com/1471-2105/8/221) in BIOCONDUCTOR suite to perform the microarray normalization. The ChIP peaks were calculated with a *distCutOff* value of 5000. The *upperBoundNull* method with a *p-value* of 0.05 was used to calculate the threshold for calculating the enriched regions. M is the log_2_ ratio of bound to total signal. From each microarray experiment, we obtained a set of enriched regions defined by chromosome number, start, stop, maxLevel, and score of each peak. For experimental triplicates, all nucleotides were examined to identify those enriched in at least two of the replicates. Nucleotides pertaining to contiguous stretches of enriched nucleotides ≥500 bp were identified. Finally, these enriched regions were eliminated if 50% or more of their nucleotides overlaps with enriched nucleotides in the control datasets. The remaining enriched regions are deemed “bound”.

### Analysis of Intersection between Datasets

Bound regions from different datasets that overlap by ≥100 bp were deemed to intersect and were enumerated within the intersecting region of the Venn diagrams. Details on set functions and construction of the Venn diagrams are described in **Methods S1**.

### Calling Fkh1-only, Fkh2-only, and Fkh1and2 Sets

Fkh1-only loci were defined as the union of Fkh1-dependent and Fkh1-Myc loci followed by subtraction of Fkh2-Myc loci. Fkh2-only loci were defined as the union of Fkh2-dependent and Fkh2-Myc loci followed by subtraction of Fkh1-Myc loci. Fkh1and2 loci were defined as all loci with subtraction of Fkh1-only and Fkh2-only loci. For union of sets, all nucleotides in the sets being combined were included in the union. For subtraction of a set B from a set A, enriched regions in set A were entirely eliminated from set A if they overlapped by ≥100 bp with enriched region(s) from set B. For smaller overlaps, only the overlapping nucleotides were eliminated from set A. The remaining enriched sequences of set A comprise the subtracted set.

### Analysis of Fkh1/2 Enrichment at Genetic Elements

Heat maps of Fkh1/2 binding proximal to features of interest were constructed from two-dimensional binary matrices. Each row of the matrix represents nucleotides on either side of one instance of the chromosomal element of interest; there are as many rows as there are instances of the element class under analysis. The central column (plotted as coordinate 0) represents a central reference nucleotide for each instance of that chromosomal element, and on either side are the surrounding nucleotides, with one nucleotide per column. A matrix value of 1 indicates that the nucleotide position was called as enriched in the ChIP analysis, whereas a value of 0 indicates that the nucleotide was not enriched. The average value for each column was plotted as the binding frequency. Values given in the text and figure are the maximum binding frequency within 100 bp (500 bp for Y′) of coordinate 0. Coordinates for all genetic elements were acquired from SGD, with the exception that coordinates for ACSs in Figure 5A were taken from [28].

To test the significance of enrichment of Fkh1/2 binding in the vicinity of genetic elements, we performed simulations to model the null distribution and then tested whether the actual distribution was significantly higher than the null distribution. This method was not applicable to X, Y′, telomeres, or retrotransposons because of the lack of unique sequences downstream of these elements. Details of the simulation and statistical tests are described in **Methods S1**.

### Cell Cycle Analysis of Binding

Each enriched region identified by RINGO is associated with a total score, which is a measure of enrichment across the entire region. We normalized the total score to a score per nucleotide by dividing the total score by the length of the enriched region. Next, we calculated a union set of all the enriched regions across the three cell cycle experiments (G2/M; late G1; early S), which included all nucleotides within enriched regions in any of the sets. The score associated with each enriched region in the union set was calculated as the total of the per nucleotide score of each nucleotide that belongs to that enriched region. Hence we ended up with three tracks of enriched regions with the same chromosomal coordinates, but different total scores. These three sets of total scores were subjected to k-means clustering with k = 4, and distance measure being Pearson’s correlation coefficient. Fkh1/2 binding loci that were not enriched in any of the three cell cycle experiments were excluded from this analysis.

We also assigned subsets of these union sets to genetic elements from SGD annotation file, if the enriched region overlapped with or was <100 bp from a boundary of the feature. Then we determined the class to which each feature-associated enriched region belonged and constructed the stacked bar graphs of their distribution for each genetic element. A chi-squared test was applied to the corresponding ratios of each set of Fkh1/2 binding loci associated with a particular genetic element to test whether it was significantly different from the null distribution after Bonferroni correction. The null distribution was chosen as the membership ratios of all Fkh1/2 binding loci in the four cell cycle clusters. The distributions at all individual classes of genetic elements were found to be significantly different from the null.

### Data Accession

Microarray pair files are available at GEO, accession number: GSE42567.

## Supporting Information

Figure S1 **Genome-wide analysis of Fkh1 and Fkh2 chromatin binding.** Plots show averaged ChIP-chip signal from three experimental replicates along each chromosome, with enriched regions plotted in purple. The antibody and strain genotype used for each experiment are indicated to the left of each panel. The corresponding strains from top to bottom are: CVy43, ZOy1, CVy138, CVy139, ZOy3, ZOy4, and CVy43. Triangles on the bottom panel indicate the position of determined binding sites as described in the text, color-coded by classification.
(RAR)

Figure S2 **Cell cycle binding of Fkh1/2 genome-wide.** Plots show averaged ChIP-chip signal from three experimental replicates along each chromosome, with enriched regions plotted in purple. The cell cycle arrest for each experiment is indicated to the left of each panel.
(RAR)

Table S1 **Enriched regions for each experiment performed in triplicate.** Each row gives genomic coordinates of enriched regions from data combined from ChIP-chip experiments performed in triplicate. Strain and antibody used are indicated in the key.
(RAR)

Table S2 **Genomic coordinates of Fkh1 and Fkh2 binding sites organized by class.** Enriched regions indicated for Fkh1-only, Fkh2-only, and Fkh1and2.
(CSV)

Table S3 **Genomic coordinates for Fkh1 and Fkh2 consensus sites.** Each row gives coordinates of a single Fkh1 or Fkh2 consensus site as indicated.
(CSV)

Table S4 **Genes with upstream Fkh1/2 enrichment.** Genes are listed for which the upstream region overlaps with a Fkh1 or Fkh2 enriched region. 500 bp regions upstream of transcription start sites for ORFs and snoRNA and tRNA genes acquired from SGD were analyzed for overlap with Fkh1 or Fkh2 enriched regions.
(XLSX)

Table S5 **Strain information.** Name, genotype and source of each strain used in this study.
(XLSX)

Methods S1 Additional details of methods are given along with schematics of methods used to define intersections, unions, and subtractions, as well as methods and formulas used to calculate Venn diagrams.
(DOC)
