# Supplementary material for: Fkh1 and Fkh2 Bind Multiple Chromosomal Elements in the S. cerevisiae Genome with Distinct Specificities and Cell Cycle Dynamics
Source: PLoS One. 2014 Feb 4;9(2):e87647. doi: 10.1371/journal.pone.0087647 (PMC3913637; doi:10.1371/journal.pone.0087647)
Supplement: Methods S1 — Additional details of methods are given along with schematics of methods used to define intersections, unions, and subtractions, as well as methods and formulas used to calculate Venn diagrams. (DOC) [file pone.0087647.s008.doc]

**SUPPLEMENTAL METHODS**

**Defining set operations on sets of enriched regions**

For the following schematics, Red and Blue blocks represent enriched regions from individual experiments. Yellow blocks represent intersections, while unfilled blocks represent regions excluded from intersection. Dashed vertical lines represent boundaries of regions used for determinations.

## Intersection

For two sets of peaks, we determined whether any two peaks overlapped by 100 bp or more. If so, that region was included in the intersection set and the operation was denoted by ‘.’. Shown schematically below.


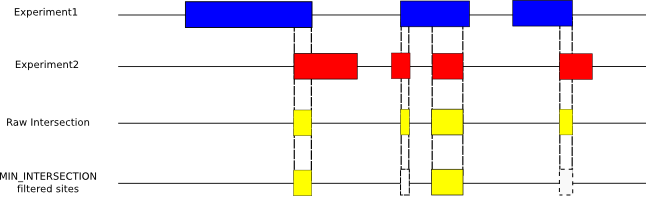


## Union

For two sets of peaks, if a nucleotide was called enriched in either of the sets, it was included as enriched in the union set and this operation was denoted by ‘+’. Shown schematically below.


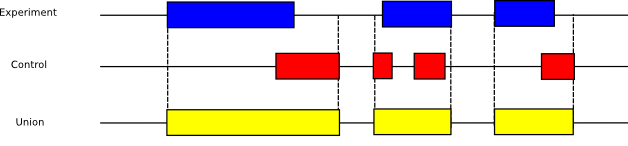


## Subtract

For two sets of peaks A and B, the subtraction operation was denoted by symbol ‘\’.

A peak in set A that overlapped by less than 100 called nucleotides in set B was included in A\B after taking away the overlapping nucleotides if any. Shown schematically below.

**
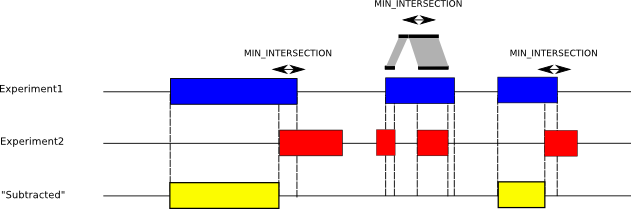
**

## Venn diagrams of enriched peaks

Creating a three-way Venn diagram can be thought of as an operation that takes three sets of enriched regions and outputs seven sets of enriched regions. In the schema below we illustrate a three-way Venn in which sets A,B,C are transformed to sets R1 through R7 which represent the seven sections of the Venn diagram. The formulas in each sector indicate how each calculation was done.


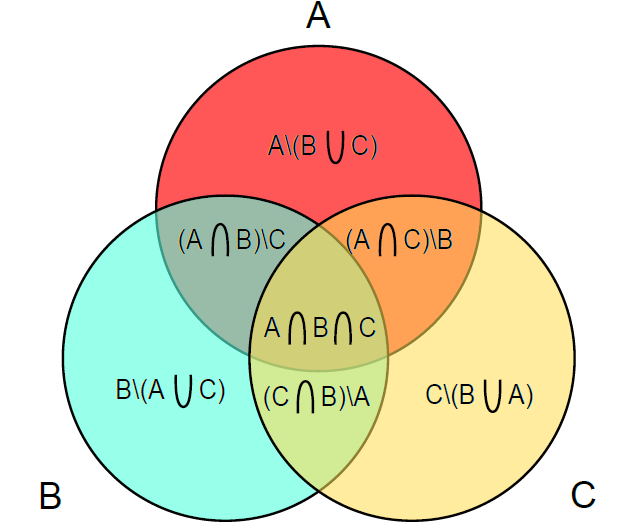


The seven sets are calculated in the following way (operations ‘.’, ‘+’ and ‘\’ defined above):

R1 = A\(B+C)

R2 = (A.B)\C

R3 = (A.B.C)

R4 = (A.C)\B

R5 = B\(A+C)

R6 = (C.B)\A

R7 = C\(B+A)

Because our sets of peaks are not discrete (i.e., enriched regions in different sets have different endpoints), there will sometimes be discrepancies in counts arising from cases in which an enriched region in one set overlaps with more than one enriched region in another set.

**Significance testing of enrichment in Mcm1 and Ndd1 binding sites**

For the Mcm1 binding sequences, which occur at known locations in the genome, we place a 1000bp window, centered on the sequence. Given that there are 79 Mcm1 sites, these windows occupy a total length of 79,000 bp (assuming non-overlapping windows) in a genome of 12,057,495 bp, a fraction of p = 0.0066.

N features are laid down independently at random across the genome, and we ask whether the number X observed to fall in the Mcm1 windows is consistent with the fraction p = 0.0066, or is consistent with the alternative that origins tend to occur more frequently in Mcm1 windows (p > 0.0066).

Thus, we use the binomial distribution to calculate a p-value for the test of p = 0.0066 versus p > 0.0066. This is calculated as Prob(a binomial with parameters N and p=0.0066 is ≥ X), which is readily computed in standard statistics packages.

The table below gives the p-values for different features for the Mcm1 sites. Those features marked with * appear to be associated with Mcm1 locations.

| Feature | N | X | P-value |
| --- | --- | --- | --- |
| Activated Origins | 58 | 0 | 1 |
| Unregulated Origins | 83 | 0 | 1 |
| Repressed Origins | 45 | 0 | 1 |
| Clb2 Cluster genes | 32 | 6 | 6.4643e-08 * |

We used a similar approach to analyze the 315 Ndd1 sites, for which p = 315,000/12,057,495 = 0.0261. The table below gives the p-values for different features for the Ndd1 sites. Those features marked with * appear to be associated with Ndd1 locations.

| Feature | N | X | P-value |
| --- | --- | --- | --- |
| Activated Origins | 58 | 2 | 0.4490 |
| Unregulated Origins | 83 | 8 | 0.0015 * |
| Repressed Origins | 45 | 2 | 0.3290 |
| Clb2 Cluster genes | 32 | 7 | 1.5628e-05 * |

**Significance testing of enrichment in the heat maps**

For each chromosome, we first placed the features of interest in their respective places.
On each chromosome, we calculated the space left behind after placing the n ChIP peaks from that chromosome (L). We generated n uniform random variables that divided the length L into n+1 segments. After permuting the order of the n ChIP peaks, we placed the ChIP peaks on these randomly generated dividers; hence, the total length of our simulated chromosome was equal to the length of that chromosome.

Next, we calculated the median value associated with the heat map within 100 bp of the feature start site. We repeated this simulation 10000 times and calculated the number of times the median of the simulation was greater than or equal to the actual value of the median. This yields a p-value indicating the significance of enrichment near the feature start. We chose a significance cutoff of 0.001.
